# Automated acoustic detection of Geoffroy's spider monkey highlights tipping points of human disturbance

**DOI:** 10.1098/rspb.2022.2473

**Published:** 2023-03-29

**Authors:** Jenna Lawson, George Rizos, Dui Jasinghe, Andrew Whitworth, Björn Schuller, Cristina Banks-Leite

**Affiliations:** ^1^ Grantham Institute, Imperial College London, UK; ^2^ Department of Life Sciences, Imperial College London, UK; ^3^ GLAM – Group on Language, Audio, & Music, Imperial College London, UK; ^4^ Osa Conservation, Conservation Science Team, Washington, DC 20005, USA; ^5^ Institute of Biodiversity, Animal Health, and Comparative Medicine, College of Medical, Veterinary and Life Sciences, University of Glasgow, Glasgow, Scotland, UK; ^6^ Department of Biology, Center for Energy, Environment, and Sustainability, Wake Forest University, Winston-Salem, NC 27109, USA; ^7^ EIHW – Chair of Embedded Intelligence for Health Care and Wellbeing, University of Augsburg, Augsburg, Germany

**Keywords:** threatened species, primate, passive acoustic monitoring, bioacoustics, automated species detector, tipping points

## Abstract

As more land is altered by human activity and more species become at risk of extinction, it is essential that we understand the requirements for conserving threatened species across human-modified landscapes. Owing to their rarity and often sparse distributions, threatened species can be difficult to study and efficient methods to sample them across wide temporal and spatial scales have been lacking. Passive acoustic monitoring (PAM) is increasingly recognized as an efficient method for collecting data on vocal species; however, the development of automated species detectors required to analyse large amounts of acoustic data is not keeping pace. Here, we collected 35 805 h of acoustic data across 341 sites in a region over 1000 km^2^ to show that PAM, together with a newly developed automated detector, is able to successfully detect the endangered Geoffroy's spider monkey (*Ateles geoffroyi*), allowing us to show that Geoffroy's spider monkey was absent below a threshold of 80% forest cover and within 1 km of primary paved roads and occurred equally in old growth and secondary forests. We discuss how this methodology circumvents many of the existing issues in traditional sampling methods and can be highly successful in the study of vocally rare or threatened species. Our results provide tools and knowledge for setting targets and developing conservation strategies for the protection of Geoffroy's spider monkey.

## 1. Introduction

The number of species threatened with extinction is increasing drastically. A recent report by the Intergovernmental Science-Policy Platform on Biodiversity and Ecosystem Services (*IPBES*) found that over 1 million species are now under threat, which impacts the ecosystems and processes they support [1]. Monitoring and evaluating the response of rare and threatened species to anthropogenic change is essential for effective management and improved decision making [2–4]. Despite decades of research, we still lack the evidence to effectively conserve the world's primates [5].

Of the 504 species of primates, 60% are threatened with extinction and 75% are declining as a result of human disturbance [6]. Primates can be difficult to study due to their affinity for residing in the canopy [7,8], reduced population sizes, sparse distributions and often elusive nature [9,10]. Data related to species ecology and conservation requirements often involve wide spatial and temporal scales [9–11], which can be constrained by practical and economic challenges [11,12]. For example, the most common approach, the line transect, requires extensive person power and establishing walking transects through dense forests remains a challenge, hindering the ability to carry out large-scale or long-term studies, which are considered key for understanding how to effectively conserve primates [5,12]. Current methods also require the researcher to be always present, potentially leading to biased behaviour and results [13,14]. Additionally, there is a risk of missing detections if both observers and animals do not happen to cross paths in a given space at the precise time of the survey. More recently camera trapping has been used to study primates [15]. Camera trapping can be more effective at identifying individuals, however, the detection space for camera trapping (e.g. a couple of metres) is much more limited when compared to acoustic monitors (e.g. hundreds of metres) and setting camera traps in the canopy is logistically challenging and expensive. The emerging field of passive acoustic monitoring (PAM) can overcome these constraints for vocally active groups such as primates. Acoustic sensors can be deployed in the field for long periods of time from just a few days, [11,16] to several months [17] and monitor continuously, without the need for the researcher to be present. In the absence of networked sensors, deployment time is only limited by battery and memory card capacity. This allows for the potential to increase the temporal extent of a study, reduce disturbance on the individuals, and increase the chance of detecting rare or elusive species [13,18,19]. The reduction of person-power required in the field, wide detection spaces (albeit species dependant due to differences in amplitude and frequency of calls), increased feasibility in challenging terrain and the increasing affordability of sensors also offers the ability to study across greater spatial scales, enabling researchers to understand the impacts of anthropogenic disturbance across much larger areas [4,20]. PAM has already been shown to be effective in the study of vocally rare species of birds [11,16], mammals [14,19] and anurans [21]. PAM offers many of the same benefits as camera trapping, however, PAM can be up to five times more effective at detecting individuals than camera traps [17].

While PAM dramatically reduces the burden to collect field data, the methods for extracting information from the recordings poses significant challenges. Large acoustic data sets are time consuming to analyse manually, requiring automated detection and classification systems to extract sounds [18,19]. The development of these tools requires specialist skills and large labelled training datasets, which are difficult to collate, especially for rare species [18,22]. Hence the creation of automated models for detecting species is recognized as a major bottleneck in the field, especially in the tropics, since most models have been created for temperate regions [18]. The lack of specific classification tools introduces additional concerns, since using pre-trained models on different environments might introduce acoustic domain mismatch concerns [23]. Automated analysis tools for primates have so far largely focused on African and Asian species [19,22,24–29], with only one model for a Neotropical species created based on calls from a small group of captive marmosets, designed for use in the medical field [30]. Classifiers for new world primates across Latin America are needed if we are to use PAM to study these species at large scales.

Deep learning (DL) is a machine learning (ML) discipline that has been revolutionary in its success in modelling high-complexity data, including in the domain of computational bioacoustics [31], in which there has been an increase in predictive performance compared to more traditional ML approaches. We opted to use DL for its capability in modelling PAM data, inspired by previous studies, such as the discrimination among calls of different primates recorded in a wildlife sanctuary [32], and the detection of Bornean [33] and Hainan [22] gibbon calls, as well as calls by Geoffroy's spider monkey (*Ateles geoffroyi*) [34] using PAM data. Here, we use the deep convolutional model proposed in [34] for acoustic call detection.

Geoffroy's spider monkey are found from south-eastern Mexico to north-western Colombia [35]. They are classified by the IUCN as Endangered and the population is predicted to decline by 50% over a 45-year period [36]. This species is a large-bodied primate with a home range of up to 4 km^2^, a frugivorous diet and requirement for large mature trees as sleeping sites [7,8,37–40]. Due to this, they require large areas of undisturbed mature forest and are therefore particularly sensitive to forest loss and fragmentation [41,42]. There have been several studies investigating how Geoffroy's spider monkey responds to anthropogenic change, revealing contradictory results regarding their ability to tolerate land use change [7,8,15,37,39,43–49]. The effects of human infrastructure have gone largely unstudied, with only one study revealing avoidance of habitat close to roads and an effect of canopy gap on crossing [50] and one showing human density to have no effect on occurrence [43]. A recent study used PAM to study Geoffroy's spider monkey in Mexico, however, detection rates were just 32%, possibly because the authors used cluster analysis rather than an automated classifier, which separates data points into similar clusters [51]. Although a simpler and more rapid approach, cluster analysis can suffer from issues related to the positioning of data in relation to other points and not necessarily the data itself, and is therefore often used when preparing the data for more intensive analysis, not as the final approach.

The aim of this study is to assess if the application of PAM and a recently developed automated detection and classification system for the spider monkey call, is effective at providing informative data on Geoffroy's spider monkey at 341 sites across a region spanning 1093 km^2^ in the Osa Peninsula, Costa Rica. We use presence and absence to assess how this endangered primate responds to habitat loss and human influence across a gradient of disturbance. Specifically, we answer the following questions: (1) is PAM effective in studying Geoffroy's spider monkey across large spatial scales? (2) How does land use change, forest cover and density of roads and human settlements affect the presence of the spider monkey?

## 2. Methods

### (a) Study site

**Figure 1.**
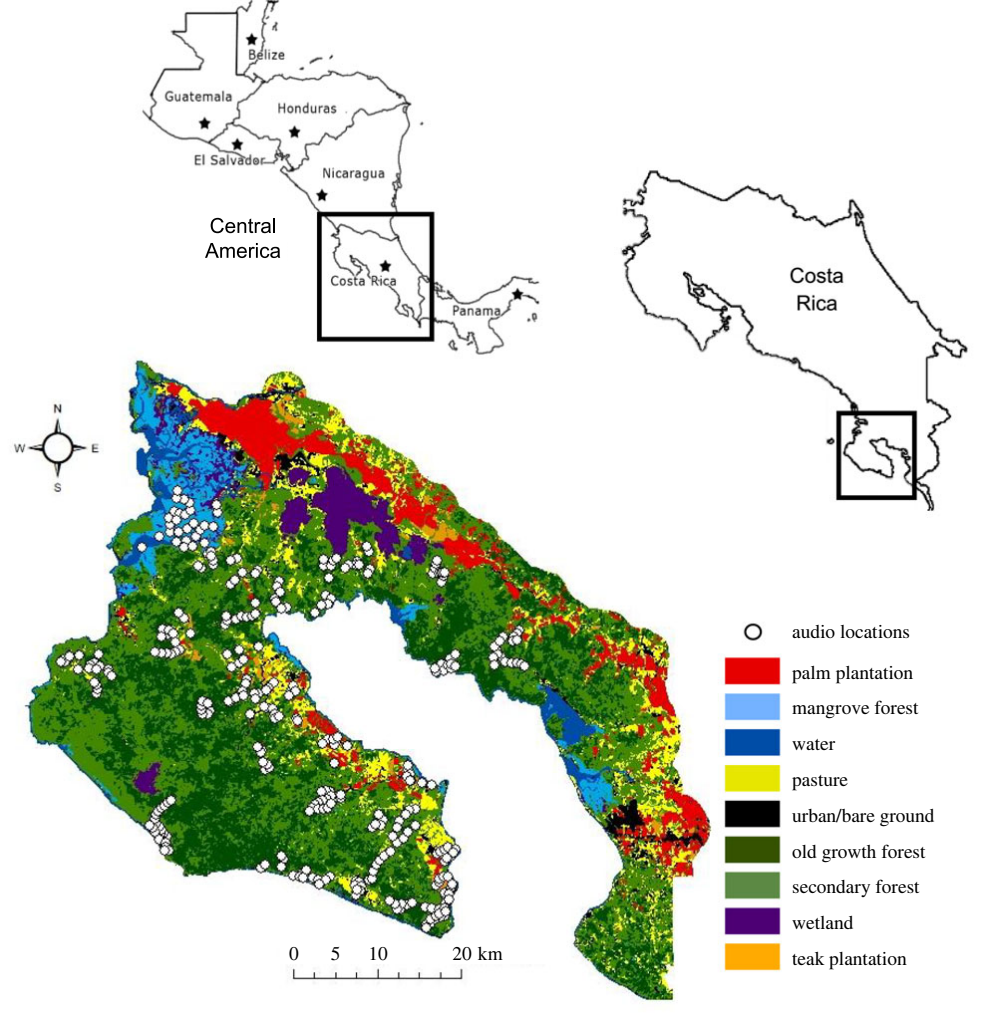
Land use map of the Osa Peninsula. Map showing the nine land use categories in the region, created at a scale of 5 × 5 m using Landsat 5 Thematic Mapper (TM) and Landsat 8 Operational Land Imager (OLI) [53]. White circles represent the sample sites where each audio recorder was placed.

Our study area covers approximately 1000 km^2^ in the South Pacific coast of Costa Rica. The terrain is generally low altitude, with a maximum elevation of 792 m. Mean annual rainfall ranges from 3000–6500 mm and mean yearly temperature is 27°C, with high levels of humidity throughout the year. There are two distinct seasons, wet and dry season, with the highest rainfall occurring September through December [52]. The peninsula contains the last remnants of tropical broadleaf evergreen lowland rainforest on the Central American Pacific [52], embedded within a mosaic of pasture, plantations and urban centres (figure 1). Managed under the Area de Conservación Osa (ACOSA), the Osa Peninsula contains three core protected areas, Piedras Blancas and Corcovado National Parks and a Ramsar wetland site, the Terreba-Sierpe Wetlands. There are also nine smaller private and public wildlife refuges and the Golfo Dulce Forest Reserve [54].

### (b) Sampling design

Habitat type at each site was calculated using land use maps provided by NASA, created at a scale of 5 × 5 m using Landsat 5 Thematic Mapper (TM) and Landsat 8 Operational Land Imager (OLI) [53] (figure 1). The map was classified into nine land use categories, with recordings for this study taken in old growth and secondary forests, palm and teak plantations, mangroves and grassland sites (figure 1). We digitized information on roads and buildings across the study site by manually annotating all primary and secondary roads and buildings in the region using satellite imagery within ArcGIS software.

We used a stratified sampling approach to ensure a representative number of sampling sites were chosen across each land use category. We calculated the percentage cover of each land use category across the region and placed a representative number of recorders in each category (see electronic supplementary material, S1.1, table S1). To guarantee even coverage of the study region, we selected sampling locations in a uniform distribution across the Osa Peninsula. Due to access issues, it was not possible to randomly choose sampling locations in all areas, therefore, to ensure independence among sampling locations, the first recorder in each area was placed by walking 500 m in a random direction. A minimum distance of 500 m was used between sampling locations to ensure independent sampling based on an average spider monkey home range size of 80 ha, which translates to a radius of 500 m [51]. We tried to avoid using trails to reduce bias, however, where this was not possible, devices were placed a minimum distance of 200 m perpendicular to a trail; as indicated by GPS. Non-audio data were collected for each point including GPS location, elevation and land use, to verify data from NASA land use maps [53]. Recording devices were also placed at a minimum distance of 200 m from habitat boundaries, to be confident that calls were from within the classified habitat. Where hotels and houses were present, we placed recorders at a minimum distance of 50 m from buildings to respect the privacy of local residents and guests and to minimize the effect of buildings on detection. Data were collected at 341 sites for 15 h per day and for 7 days, totalling 105 h per site and a total of 35 805 h of recordings. Twenty-six sites were not included in the analysis due to malfunctioning of recording equipment leading to less than 7 days of recordings. Recordings were obtained using Audio Moth devices (Open Acoustics Devices, UK). Recorders were set up for 7 consecutive days to increase the chances of detecting the spider monkeys if they were present and due to limitations in the capacity of the memory cards. The devices were set to record on a schedule of 05.00–09.30, 14.00–18.30 and 21.00–03.00, to guarantee data were collected at key periods of spider monkey activity [55]. We chose to record during the night as the spider monkey has been found to be active at night [15], and given that primates have been found to change their vocal communication in the presence of increased anthropogenic sound [56], it was important to capture periods where human activity is likely to be reduced. We recorded constantly over the recording schedule at a sample rate of 48 000 kHz, 2.5 times higher than the maximum call of Geoffroy's spider monkey. Sampling was conducted within the dry season (December–August) due to restricted access to many areas of the study site during the wet season. We installed acoustic recorders in six of the nine land use categories: old growth and secondary forest, mangrove forest, grassland, palm and teak plantations.

### (c) Automated system for signal classification

Animals often use several different calls for communication; however, it was not feasible to study all 13 calls of Geoffroy's spider monkey [57]. During six weeks of recording in areas where the species was known to be present, we found that over 80% of recorded calls were the ‘whinny’ and every calling period contained several examples of this call type. The whinny represents general communication related to feeding and movement [58]. This call was therefore chosen for use in the classification algorithm [34].

#### (i) Residual convolutional neural network

Deep convolutional neural networks learn from large amounts of annotated data to make the desired predictions, by having each convolutional layer learn and perform a progressively more complex nonlinear feature transformation, rather than requiring major prior feature engineering [59]. They are designed to process two-dimensional data, like images, or, as in our case, spectro-temporal audio representations like the log Mel spectrogram [60]. The final process of classification provides a set of confidence scores about how likely the sound is to belong to a particular class [59].

The classifier used in this study for whinny detection was first proposed in [34] as an improvement upon a deep, convolution-based, neural network architecture for acoustic event detection [61]. This improvement was achieved via the addition of attention-like mechanisms [62] that learn how to apply importance weights to the also learnt features as described in detail in [34]. Specifically, the model uses a squeeze-and-excitation mechanism [62] after each convolutional layer to reweigh the outputs of the convolutional filters, as well as a multiple-head attention mechanism [63] for pooling the sequential audio representation into a single, fixed vector representation.

We follow the optimization approach of [34] for training the model, using the same dataset partitions which are site-independent in order to make sure that the model does not depend on learning site-dependent acoustic characteristics in the validation process. The model was implemented using the Tensorflow (v. 1.15) Python library [64]. The model was trained on data obtained from 13 sites and recorders. We manually listened to 600 h of acoustic data from these sites and isolated 591 examples of the target sound in a total of 366 sound files. Data for creating the training dataset was taken in old growth and secondary forests. We could not train the model on data from other land use types as we did not detect the spider monkey in other land use types during out pilot study, however some recording sites were bordering more disturbed land use types such as palm and teak plantations.

#### (ii) Model validation

Here we report recall and precision of the test partition, which constitutes data from three sites, in an unweighted average manner: this means we average the respective values for the positive and negative class. Model results showed that unweighted average recall was 75% when a confidence threshold of 50% was set. As we were using a semi-automated approach, we manually checked all returned positives, and then used these results to build up a database of calls. Precision was a little lower at 53%, indicating that the model was making some incorrect classifications, i.e. false positives and false negatives. The F1 score was 62%, which takes into account precision and recall, providing an overall estimate of model accuracy. This performance profile, with high recall, even at the cost of some precision, is appropriate for our purposes since we are using a semi-automated approach.

**Figure 2.**
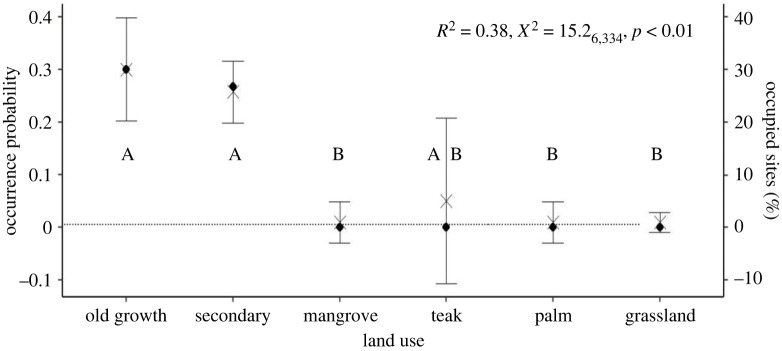
Land use model results. Probability of species occurrence in each land use type on the *y*-axis (X), error bars represent 95% confidence intervals and percentage of occupied sites in each land use type on the *z*-axis. Pairwise differences between land use are indicated with letters A and B, where different letters represent a significant difference between land use. *R*^2^, *X*^2^ and *p*-values are annotated on each plot.

Whereas other DL bioacoustics studies on primates have achieved higher unweighted average recall on their respective datasets, we believe our seemingly comparatively lower performance is justified for two reasons. Firstly, compared to the study performed by Pellegrini [32] on recordings from fenced areas in a wildlife sanctuary, our data are truly collected in the wild, meaning that there is a greater variance in both the types of potential calls of other animals that can be present, and the potential distance of the animals from the recording device. This means that our dataset contains some hard positive samples, where the call is faintly audible, as well as negative samples in which other calls are audible, but are either from other species, or are not specifically the whinny (see electronic supplementary material S1.7. (figure 2) for example spectrograms). Secondly, compared to the gibbon-focused studies in [33] and [22], we have fewer positive examples for training, which impacts the performance of DL techniques [31]. That being said, even though studies like Dufourq *et al.* [22] mention that in such cases we should typically use smaller and shallower model architectures in order to avoid overfitting on the limited training set, Rizos *et al.* [34] instead showed that it was the deeper and more complex models that achieved the highest performance (compared to models like the ones used in [22,33]), including the one we use in this study.

In order to investigate the performance of this model in further detail, we also report its performance on two subsets of the test partition where we knew how many positive calls were contained in the files, tested by one team member experienced in detecting and classifying the calls of *A. geoffroyi*. This information can be found in electronic supplementary material, S1.2. Using the best model above, we then ran data from all 341 sites through the model algorithm using a confidence threshold of 50%. The model outputs all positives into .csv files and an associated folder with clipped audio files. We listened to all returned audio files and marked the number of true positive detections per day per site. All analysis was carried out in Python v. 3.6 [65].

### (d) Statistical analysis

#### (i) Explanatory variables

To correctly assess species responses to habitat changes, it is crucial that predictors are measured at the scale at which the species responses are strongest, known as scale of effect [66,67]. To assess the scale of effect of forest cover, primary (paved) and secondary (unpaved) roads and buildings, we calculated percentage forest cover (%), the density of roads (km) and the area of buildings (km^2^) in a buffer around each site at intervals between 100 and 5000m radius for forest cover and 100–1000 m for roads and buildings (see electronic supplementary material, S1.3. table S2). We then tested at which spatial scale the variables had the strongest effect on the spider monkey. The range of spatial extent chosen allowed us to investigate occurrence resulting from interactions at a local level (within the immediate surroundings) and the dispersal of species, factors that are related to habitat selection [68]. It has been demonstrated that overlap between buffers does not increase spatial autocorrelation in model residuals, thus not violating model assumptions [68,69].

The data showed complete separation across land use and primary road variables, which happens when a combination of the explanatory variables produces a perfect prediction of the response variable [70]. This led to high standard errors, confidence intervals and *p*-values. To account for this, a model containing these variables was fitted separately in package *brglm2* [71] that can model complete separation*.* We fitted all other models in the *nlme* [72] and *lme4* package [73].

We included the variable month as a random effect to determine whether seasonality influenced detections. Its inclusion did not improve model performance based on AIC and log-likelihood results, (electronic supplementary material, S1.4. table S3), model coefficients and results remained the same and explained variance in presence as seasonality was low (*R*^2^ = 0.013) (electronic supplementary material, S1.4. table S4). Month as an explanation of seasonality was therefore not included in the final model.

#### (ii) Generalized linear models

Data for each site consisted of 7 continuous days of recordings. A detection period constituted one 24 h period of recording, providing a 7-day detection history per site. Each day was coded as 1 or 0 for presence or absence. Site occurrence was calculated by combining the 7-day detection histories for each site into one parameter. Presence on 1 or more days was coded as 1 and absence on all days was coded as 0. To determine the probability of occurrence of the spider monkey across our study site, we used logistic regression with a logit link function. Although there was some correlation between variables, it was generally low (*R*^2^ = 0.15–0.27) with the exception of buildings and primary road variables (*R*^2^ = 0.58) (electronic supplementary material, S1.5, figure S1), however, these were run separately due to complete separation in the primary road data. Primary road and land use were significantly correlated (*p* < 0.01) and hence, within package *brglm2,* these variables were run separately. Bonferroni's correction for multiple pairwise comparisons was applied to adjust *p*-values and reduce the risk of type I errors.

We ran variance partitioning analysis in package *hier.part* to understand how much of the variance explained by each explanatory variable is individual or shared (i.e. cannot be ascribed separately to any one variable). We used residual plots to assess violations in model assumptions. All plots showed no deviation from the expected distribution or heteroscedasticity in the residuals.

#### (iii) Spatial autocorrelation

Due to the nested structure in the data and due to the assumption that species distribution is expected to be limited to certain regions, we tested for spatial autocorrelation across all generalized linear models. We created a distanced based weight matrix using model residuals and sampling site coordinates. Using the *gstat* package [74] we then produced a variogram plot to visually determine the presence of autocorrelation. Finally, we calculated the Moran's I statistic in the *ape* package [75]. If no autocorrelation is present, the observed autocorrelation should be close to 0 and to the expected value.

Spatial autocorrelation was found in the residuals across all models, violating the spatial independence assumption of regression analysis and risking a type I error. To account for this, we constructed a spatial auto-covariate that was included as an additional predictor variable. For each site we calculated a distance-weighted average of neighbouring response values, using a minimum neighbour's distance of 210 m, with sites further away receiving lower weightings [76]. To test whether the auto-covariate function reduced autocorrelation in the residuals we used Moran's I statistic. No autocorrelation was present across the models when the auto-covariate function was added (electronic supplementary material S1.6, table S5). We also used the likelihood ratio test and AIC to determine if the addition of an auto-covariate function improved model fit. In cases where the auto-covariate function reduced autocorrelation but model fit remained equal, it was still included in the model (electronic supplementary material, S1.4, table S3)

#### (iv) Occupancy

We initially explored the use of occupancy analysis to control for imperfection detection. Our null model, where we assumed that detection probability and site occupancy were constant across time and space and no covariates were included, showed occupancy to be similar to naive estimates (0.187 versus 0.192). We constructed a set of candidate models where occupancy was modelled as a function of environmental covariates, however, models did not converge when variables such as primary roads and land use were included due to complete separation in the data. Given the similarities in naive estimates and non-convergence of some models, we only report these results in electronic supplementary material, S2.0. All statistical analysis was carried out using R [77].

## 3. Results

The automated detection and classification algorithm for the spider monkey whinny returned a total of 2977 true positives across 273 days in 64 out of 341 sites and 52 248 false positives. To listen to this data manually it would take 20 years, on a schedule of 8 h per day, 365 days of the year. It took eight weeks to pass all 341 sites through the algorithm and to identify all true positives using a semi-automated approach. A map detailing in which sites Geoffroy's spider monkey was present is included in electronic supplementary material, S3 (figure 1).

### (a) Scale of effect

The response of Geoffroy's spider monkey to forest cover was strongest at 200 m radius (*R*^2^ = 0.77), although the response remained > 0.4 until a radius of 1000 m, highlighting that forest loss is affecting the spider monkey at a local scale and its dispersal ability. Forest cover appears to have a nonlinear relationship with response the variables, therefore a polynomial term was added to the models. For secondary roads, the strongest response was at 200 m radius (*R*^2^ = 0.09), however all spatial scales showed a similar response (mean *R*^2^ = 0.07, s.d. = 0.01) and for primary roads, the strongest response was at 1000 m radius (*R*^2^ = 0.6). For buildings, the strongest response was also at 1000 m radius (*R*^2^ = 0.23) (electronic supplementary material, S1.2, table S2). Thus, from here on, all results are presented where each explanatory variable was measured at these scales.

### (b) Land use

Land use has a significant effect on the presence of the spider monkey (figure 2), with spider monkeys only found in old growth and secondary forests. The probability of occurrence was significantly lower in grassland, palm and mangrove when compared to old growth and secondary forests (figure 2; electronic supplementary material, S3.1, table S1). We registered no records of the spider monkey in teak plantations, however, this difference was not significant due to high standard errors and confidence intervals from model fitting. Probability of occurrence was not significantly different between old growth and secondary forests (figure 2 electronic supplementary material, S3.1, table S1).

### (c) Human development

**Figure 3.**
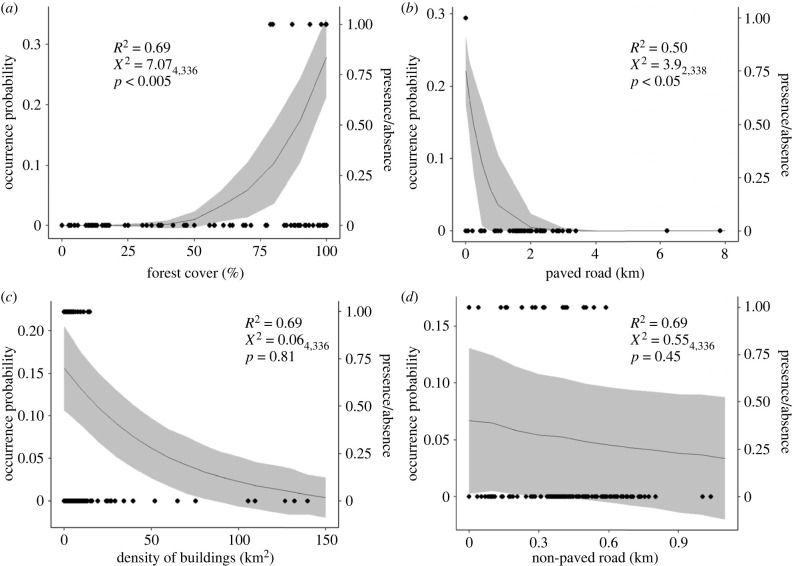
Continuous variables model results. Probability of occurrence on the *y*-axis and observed presence/absence on the *z*-axis (l) in relation to (*a*) forest cover (%) measured within 200 m. (*b*) Density of primary roads measured within a 1 km radius of the site, where what appears to be a single point denoting presence relates to multiple points. (*c*) Cover of buildings measured within a 1 km radius of the site. (*d*) Density of secondary roads measured within a 200 m radius of the site. Shaded areas represents 95% confidence intervals. *R*^2^, *X*^2^ and *p*-values are annotated on each plot.

Spider monkeys were strongly associated with higher levels of forest cover, only being found above 80% cover (*p*-value < 0.005, figure 3*a*; electronic supplementary material, S3.1, table S2). Primary roads had a negative effect on spider monkey occurrence (*p*-value < 0.05, figure 3*b*; electronic supplementary material, S1.3, table S5). The spider monkey was not found at any site with a primary road within a 1 km radius (figure 3*b*). Total area of buildings had a negative non-significant effect on spider monkey occurrence and the spider monkey was not found where cover of houses exceeded 18 km^2^ within a 1 km radius of the site (*p*-value = 0.81, figure 3*c*; electronic supplementary material, S3.1, table S2). The effect of secondary roads on spider monkey occurrence showed a non-significant decrease (*p*-value = 0.45, figure 3*d*; electronic supplementary material, S3.1, table S2). Despite this, the spider monkey was only found where the density of secondary roads was below 0.6 km within a 200 m radius of the site (figure 3*d*).

### (d) Variance partitioning

Variance partitioning showed the influence of secondary road density and area cover of buildings on spider monkeys were mostly attributed to other variables, over 65% and 50% shared variance respectively. However, forest cover (34.1%), land use (28.4%) and primary road density (31.4%) had large unique contributions, showing they are the main drivers of changes in occurrence (electronic supplementary material, S3.2, table S3).

## 4. Discussion

In this study, we have shown that PAM combined with a semi-automated detection and classification system for extracting calls can be successfully used to detect and assess how a rare and threatened vocal species, Geoffroy's spider monkey (*A. geoffroyi*), responds to changes across the landscape at a wide spatial scale. Our analysis of 35 805 h of data across 341 sites shows that this species does not occur below a threshold of 80% forest cover and is absent from areas within 1 km of primary paved roads. By contrast to what some studies have suggested [8,37,39,43,44], we found equal occurrence in old growth and secondary forests and a limited tolerance of human development.

### (a) Application of passive acoustic monitoring

The automated detector used in this study was able to analyse over 35 805 h of data in just eight weeks, which would not have been possible using manual methods. We returned a total of 2977 calls across 64 out of 341 sites. Despite a low naive site occupancy estimate of only 18.7%, using occupancy modelling to account for imperfect detection provided an estimate of 19.2%, suggesting that our estimate of occupied sites using calls extracted by the automated detector are very accurate. This is likely because there were no instances when we only detected a single call, generally, in sites where we detected the spider monkey, we detected dozens of calls over a few days. Detection probability from occupancy modelling was 68% and unweighted average recall from the automated detector was 75%, which means we have potentially a percentage of calls and therefore may be underestimating call rate, however, we set call confidence at the inclusive value of 50% to increase the number of calls returned and avoid missing true positives. Because model precision was only 53%, meaning that the model returned a significant number of false positives, we used a semi-automated approach, where we manually confirmed all positives returned by the model, which was still a very time-efficient process compared to fully manual alternative or conducting line transects to collect data. Site occupancy would have been artificially inflated if we had not taken this approach, with all sites showing false positives, severely biasing the results and affecting conservation and management recommendations.

### (b) Geoffroy's spider monkey: response to land use change

Geoffroy's spider monkey occurrence was at its highest in areas with over 80% native forest cover. Identifying thresholds, or ‘tipping points’ below which biodiversity may decline, is essential in the design of conservation strategies to prevent local extinction of species [78]. Previous research on this species in Mexico shows that they were present in areas where forest cover was above 50%, being locally extinct below this threshold [47]. It is difficult to pinpoint the exact reasons for these differences in sensitivity to changes in forest cover; however, it is beginning to emerge that there is a large intraspecific variation in responses to habitat changes and that macroecological factors may modulate populations' responses [79]. Previous work to identify thresholds of forest cover for communities in the Amazon and Atlantic rainforests in Brazil highlighted a 30–40% forest cover threshold to preserve the integrity of vertebrate communities [80–82]. The reason that the requirements are so much higher for Geoffroy's spider monkey is likely due to their specialized diet of mature fruits and requirement for mature sleeper trees [8,37,39,43,44]. In a study of African bird species, declines in overall richness were seen below 42% forest cover, however, for species with more specialized diets, diversity started to decline once forest cover was below 74%, suggesting specialist species require higher thresholds of forest cover [83]. Although reported community-based thresholds are lower, it is recognized that higher thresholds may be needed in the tropics to protect the most endangered species [84].

In this study, occurrence probability was similar across old growth and secondary forests, as found previously in the same region [7]. However, studies conducted in other regions have found that spider monkeys generally prefer continuous tracts of old growth forests [8,37,43,44], and occur in secondary forests at significantly lower levels [44,45]. The reason for the disparity in these results is likely due to the definition and characteristics of secondary forests, which may vary across studies since the term secondary forest can be used to describe forests of varying age. Owing to the protected status of forests in Costa Rica, secondary forests are generally 30 years+ [15], and the land use maps used in this study defined secondary forests as 40 years+ [53]; therefore secondary forests, as defined here, may be considerably more mature than forests in other studies. It is also possible that high levels of hunting in more accessible secondary and fragmented forests in other study regions, which reduce population densities of Geoffroy's spider monkey [82,85], do not exist to the same levels here since the spider monkey is not the main target species in our study region.

Previous studies have found use of shaded coffee (*Coffea* spp.) and cacao (*Theobroma cacao*) plantations by Geoffroy's spider monkey; however, this was only where plantations had a structure and spacing suitable for locomotion and when shaded with native forest, providing a diversity of mid and upper canopy structures and species for feeding, shelter, protection and resting [48]. They have also been found to use live fences of mature trees [40,48]; however, these studies suggest that they are only used as steppingstones to other, more favourable habitats. Studies of Geoffroy's spider monkey rarely sample non-native forests, likely due to time and economic constraints of previous sampling methods, therefore there is limited evidence for their use. In our study we did not find use of non-native forest habitats, grasslands or forestry plantations, suggesting that these habitats are not suitable for permanent or temporary use. This is likely due to the palm and teak plantations in the region having a much lower floral diversity than coffee and cacao plantations, highlighting the importance of planting native species in forest plantations to improve human-disturbed environments for wildlife. Studies of this species in mangrove ecosystems are also rare; however, use of mangroves have previously been found [86,87]. In our study, however, we did not find them in this habitat.

A strong effect of paved roads has been previously found for mammal species, owing to increased gap width and heavier traffic volume [88–92], alteration of roadside vegetation structure [93], secondary road development and increased human presence [94]. Geoffroy's spider monkey has previously been found to cross both paved and unpaved roads to a similar degree in the north of Costa Rica, however only where canopy opening was small enough to facilitate locomotion [50]. In our study they were not found at any site with primary (paved) roads within a 1 km radius. Results from variance partitioning show that very little of the variance attributed to this variable is shared, further highlighting the impact of primary roads on this species. This is the first time such an effect has been shown and provides further evidence as to the sensitivity of this species to human disturbance. The use of PAM in this study allowed us to cover a large enough area with enough sampling locations to reveal such an effect.

Density of secondary roads and human settlements were not found to significantly affect the probability of occurrence. Despite these results Geoffroy's spider monkey was only found in areas with limited levels of unpaved roads and buildings, suggesting that they cannot tolerate areas with high human development. Previous research in this area related to *Ateles* is lacking, with only one study on the effects of roads, where avoidance of unpaved roads was also found [50] and two studies related to human population size or buildings, where no separate effects were found [43].

The vocal communication of primates has been found to change in different habitats [56,95]. Gibbons (Hylobatidae) were found to call less in disturbed areas and increase their call rate at quieter times of the day [56]. It is possible that the lack of detection in more disturbed areas is due to the spider monkey altering the rate, frequency, amplitude or type of call; however, we mitigate any potential effects of this by recording at multiple times of the day, including periods where human presence would be significantly reduced or absent. As we did not find the spider monkey in disturbed areas during PAM studies or while we were working in the sites, it is not possible to test if they were present but with altered vocal communication and future work should focus on testing this hypothesis.

### (c) Study limitations, wider context and conclusions

Our results corroborate previous research showing that Geoffroy's spider monkey is highly sensitive to anthropogenic changes. We have shown a requirement for over 80% of forest cover and avoidance of any paved roads within 1 km, highlighting the dangers of forest loss and paving roads through important habitat.

While we did not use other sampling approaches to ground truth the findings of our study, our semi-automated approach ensured that all returned positives from the automated classifier were correctly classified. Traditional sampling approaches, such as point counts and line transects, are time-consuming and were found to produce lower detection rates (i.e. the rate at which an animal is detected when they are present) when compared to PAM for this species in a recent study in Mexico [51]. Hutschenreiter *et al*. recommended the use of PAM to study the spider monkey over traditional sampling approaches, with the caveat that that DL approaches should be used to improve on the detection rates achieved in their study of 32% [51], which is what we have achieved in this study. Additionally, while using multiple methodologies would have allowed for a comparison of different approaches, it would have led to a lower spatial scale and coverage, which has recently been defined as essential for the development evidence-based strategies to effectively conserve primates [5].

Primates are under significant threat from anthropogenic disturbance [6] and would benefit from more efficient monitoring methods to improve management and decision making, yet studying primates in forested environments can be difficult [7,8]. Here we have shown how PAM, combined with a semi-automated detection and classification system, has proven to be an effective method for studying vocal species within this group and has identified critical tipping points of human disturbance, which will prove valuable for setting targets and developing conservation strategies.

## Data Availability

Data and code can be found on Zenodo at https://doi.org/10.5281/zenodo.6511837 [96]. Since the raw data contains several terabytes worth of audio data, csv files are included that show the processed data and the associated code to run these data for the analysis presented. The data are provided in the electronic supplementary material [97].
